# Ka-band planar slotted waveguide array based on groove gap waveguide technology with a glide-symmetric holey metasurface

**DOI:** 10.1038/s41598-021-88054-5

**Published:** 2021-04-22

**Authors:** Luis Fernando Herrán, Astrid Algaba Brazalez, Eva Rajo-Iglesias

**Affiliations:** 1 Department of Signal Theory and Communications, University of Oviedo, Gijón, Spain; 2 Ericsson Research AB, Gothenburg, Sweden; 3 Department of Signal Theory and Communications, University Carlos III, 28911 Madrid, Spain

**Keywords:** Engineering, Electrical and electronic engineering

## Abstract

The design of a planar slot array in groove gap waveguide technology implemented by glide-symmetric holes as Electromagnetic Band Gap structure is here presented. Despite the advantages of using holes instead of pins in terms of manufacturing simplicity and cost,the larger size of the holes compared to pins needs to be considered when designing slot arrays without grating lobes. A 1 to 4 corporate feed network is designed using this technology as well. Corrugations are included to further reduce the grating lobes. Experimental results support the viability of the proposed concept for designing glide-symmetric planar arrays of any size.

## Introduction

The use of metasurfaces as part of innovative antenna designs is nowadays a reality^1–3^. Just to mention some examples, metasurfaces are used to modulate surface/leaky waves^4^ creating antennas with incredible performances in printed technology, or they are an essential part of the gap waveguide technology^5^, an alternative technology to classical waveguide technology.

The groove gap waveguide is a version of gap waveguide technology that shows features with potential to overcome the problems of conventional rectangular waveguides^6,7^. The main advantage of groove gap waveguide is the possibility of being manufactured in two pieces that do not need to be in good electrical contact when assembled afterwards. This allows the design of complex circuits by texturing one layer and using a simple metal lid placed on top. At the same time, all the advantages of rectangular waveguides in terms of low losses (this is the version of the gap waveguide technology with lower losses^8^) and power handling capability are preserved.

**Figure 1:**
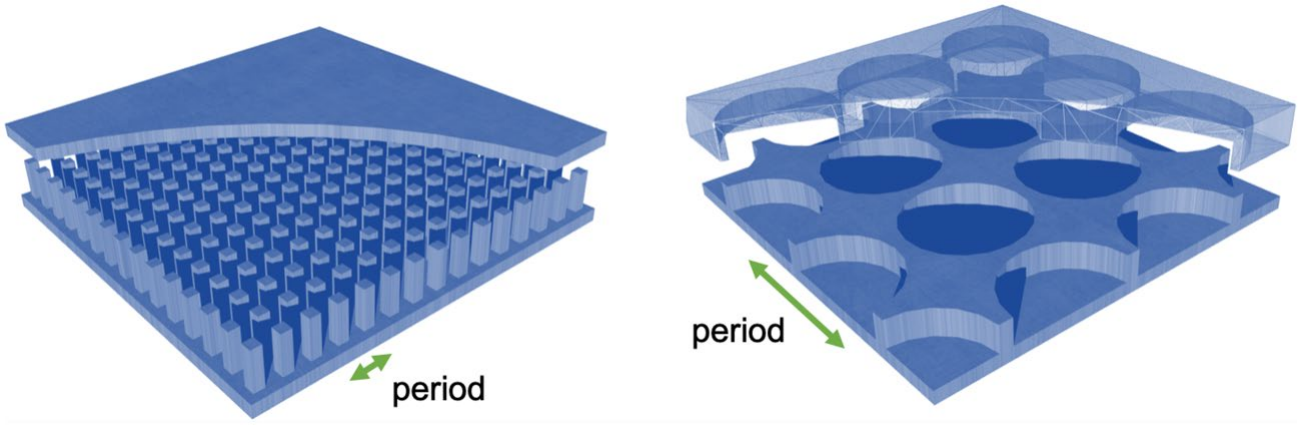
Example of comparison in size of pins and holes used to operate in the same frequency range.

Typically the metasurface used in gap waveguide technology is the known as bed of nails, i.e. metallic pins. Recently, the convenience of replacing the pins present in gap waveguide prototypes by periodic structures with simpler types of unit cells has deserved attention among researchers. One proposed option is the use of half-height pins^9^ to reduce the mechanical fragility due to the height in very high frequencies where pins can be really tiny. Another alternative based on the use of periodic holes in groove gap waveguide has been proposed^10–12^. To be rigorous, the holes need a glide-symmetric disposition in two planes to exhibit an entire and wide bandgap in all directions in a plane, otherwise the stop band only happens in one direction as in^13^. Once this is made, the width of the stopband is comparable with the one obtained with pins whilst the manufacturing is simplified. Drilling holes is way easier that milling pins and furthermore, the holes are much larger in size and periodicity when compared to the equivalent pins, which is an advantage in terms of manufacturing tolerances. An illustration of the relative sizes of holes and pins designed for the same frequency band is presented in Fig. 1.

Designing planar arrays has been one of the envisioned applications of gap waveguide technology from its original conception, including the ones based on the use of groove gap waveguide^14–20^. However, with the proposed version using holes, only a recent example based on the use of mode converters^21^, a simple linear array of horns^22^ and leaky wave antennas^23,24^ have been presented applying this technique. Some other microwave components have been proposed as presented in^25–27^. All these designs only involved one waveguide in the structure and, consequently, they are insensitive to the limitations concerning the use of an EBG consisting of a bigger unit cell that the implementation of groove gap waveguide may impose^28^. This paper presents the first example of design of a slot array antenna based on the use of groove gap waveguide technology where holes instead of pins are used. The challenge to design a power divider with this version of the technology and the flexibility of the design of the array, that is not limited to a given number of slots and has potential to be extended to a higher number of rows are some of the innovative aspects of this work.

In this work we propose the complete design of a planar slot array with a corporate feed network, based on groove gap waveguide technology implemented with glide-symmetric holes.

## Considerations on the design of waveguides with holey EBG structures

A good design of a planar slot array that is based on using a slotted waveguide as a row of the array implemented with the considered version of groove gap waveguide, starts by selecting a waveguide with dimensions as narrow as possible at the operating frequency, to allow some space for the holes that are used as EBG structure and separate the slotted waveguides. Non standard dimensions can be used for the rows of the array since there will be a power divider between the waveguides and the the input port. The input port is the one that must have the standard dimensions.

In the proposed design we use a modified version of the standard WR-28 waveguide where *a* is 6.5 mm instead of 7.112 mm, and we keep *b* as the standard value 3.556 mm. In this way only a smooth transition in the horizontal side will be needed.

**Figure 2:**
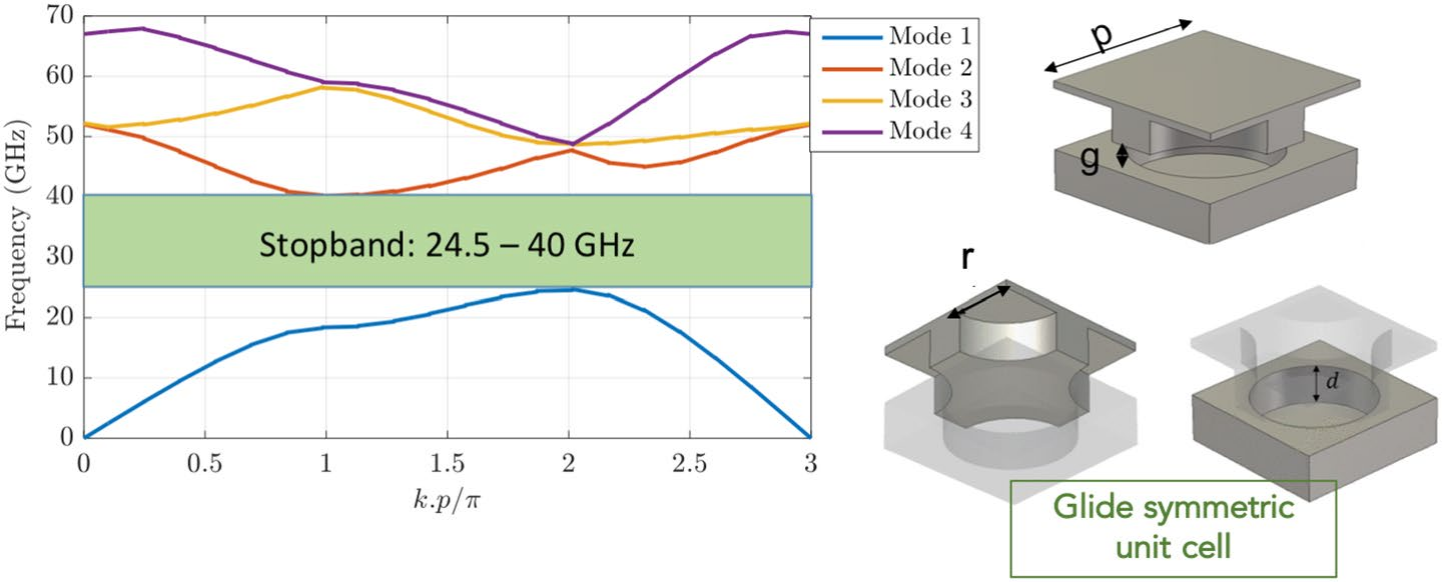
Dispersion diagram of a holey glide-symmetric EBG structure with dimensions: period $p = 4.5$ mm, hole radius $r= 1.5$ mm, depth of the holes $d= 2$ mm and an air gap of 0.1 mm.

A frequency of 28 GHz ($\lambda _0=12$ mm) is assumed as operation frequency due to its interest for the new 5G communication systems. The glide-symmetric periodic structure is designed taking into account all these constraints i.e., we select an inter-row distance smaller than $\lambda _0$. As a consequence, there is a maximum space of 5.5 mm to allocate the periodic holey EBG. We assume 4.5 mm as the value of the periodicity for the EBG and we set the other dimensions as radius of the holes $r= 1.5$ mm, depth of the holes $d= 2$ mm and a gap of 0.1 mm. The dispersion diagram of this periodic structure together with the description of the employed unit cell is illustrated in Fig. 2, where a stopband covering the desired 28 GHz frequency can be observed.

## Feed network design

**Figure 3:**
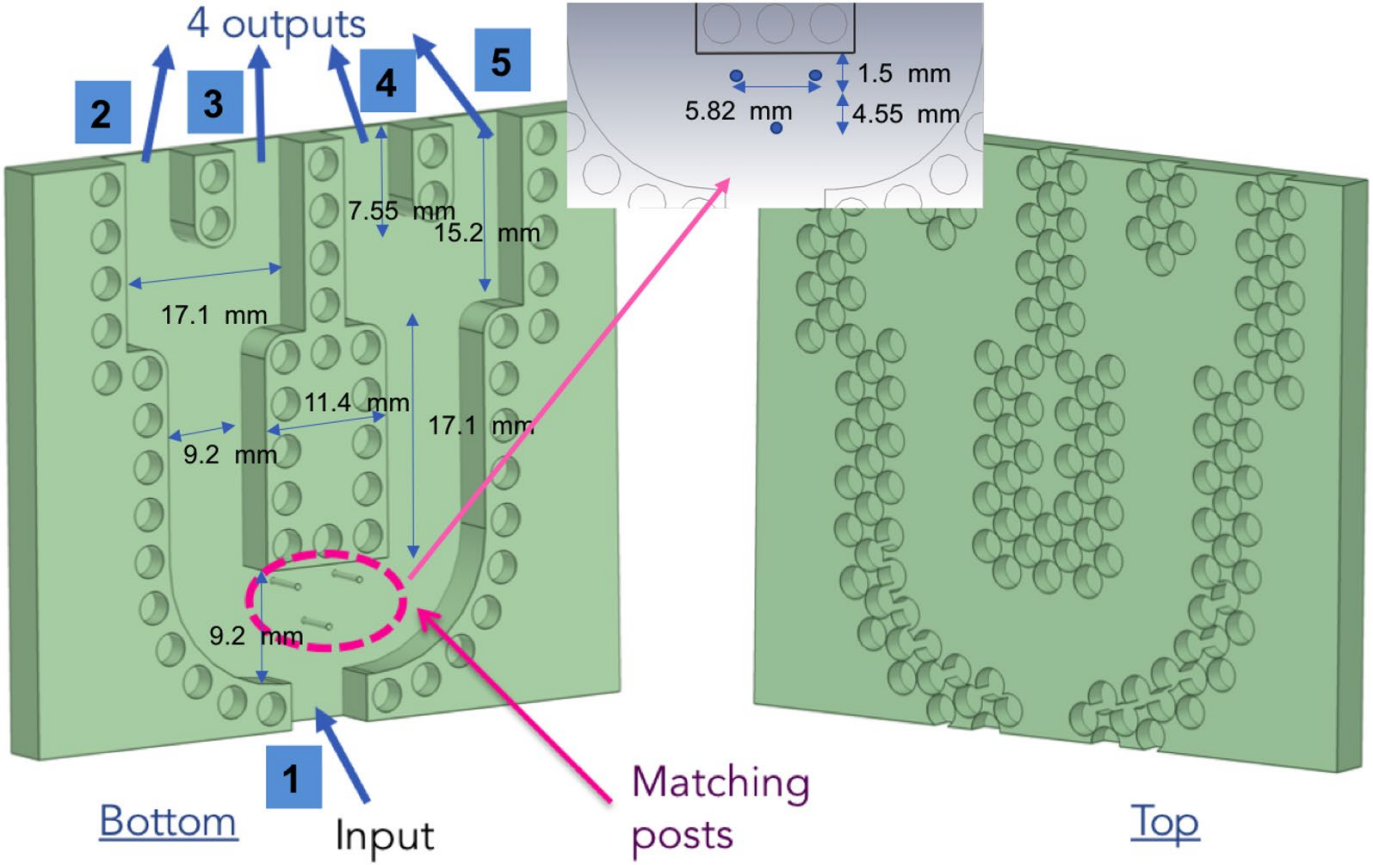
Description of the 1 to 4 power divided implemented in groove gap waveguide implemented with glide-symmetric holes.

Using the same holey structure as the described one in the previous section, a 1 to 4 corporate feed network in groove gap waveguide has been designed. The feed network is based on the use of Y dividers^29^ and the design is optimized to be as compact as possible. As key aspects of the proposed design, the use of three inductive posts at the input was chosen to be able to match these kind of power dividers^30^ (seen in Fig. 3). These posts can be also used to align the two building blocks of the prototype. The main dimensions of the power divider are described in Fig. 3.

As a particular requirement for the power divider, the input port (waveguide) must have the dimensions of a standard WR-28 to simplify the feeding of the structure, whilst the four outputs should have the modified dimensions (only the wide side) of the waveguide that will be used in the array design.

**Figure 4:**
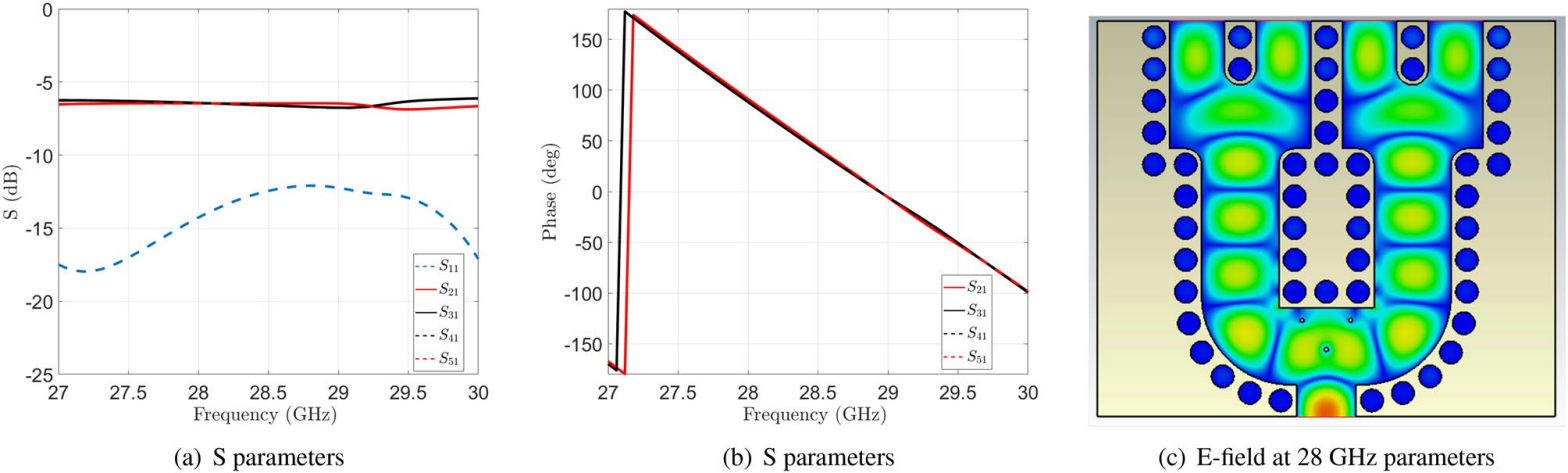
S-parameters of the simulated power divider described in Fig. 3 and instantaneous E field in the cross-section.

The feed network is described in Fig. 3 where the top and bottom pieces are represented. The simulated S- parameters for this power divider can be seen in Fig. 4. A good matching is obtained in the entire frequency range together with almost identical levels at the outputs with just a slight imbalance between contiguous ports of maximum 0.4 dB. The phases of the four output ports are also included in Fig. 4. In the same figure, the instantaneous field at 28 GHz in the middle of the waveguide is plotted. The equal division of the field in amplitude and phase can be clearly observed.

## Antenna design

Considering the dimensions of the waveguide designed in Sect. 1, a 9 element linear slot array is designed with the standard method to feed the slots with uniform distribution to create one row of the planar array. The size of the slots is 0.27 mm width by 5.09 mm long and the inter-element distance is 9.45 mm and the distance of the slots to the center of the waveguides is 0.435 mm. Considerations about how to design slot arrays in groove gap waveguide technology have been given in^16^. The starting point for the design is a conventional rectangular waveguide, and afterwards the positions are adjusted to consider the slight differences in the guided wavelength between the standard and the groove gap waveguide^7^.

The design of the individual row is made including the lateral walls implemented with the proposed holey EBG structure. These rows will be separated from the contiguous ones by the EBG structure and a total of four rows will be used to be connected to the designed power divider presented in Sect. 2.

**Figure 5:**
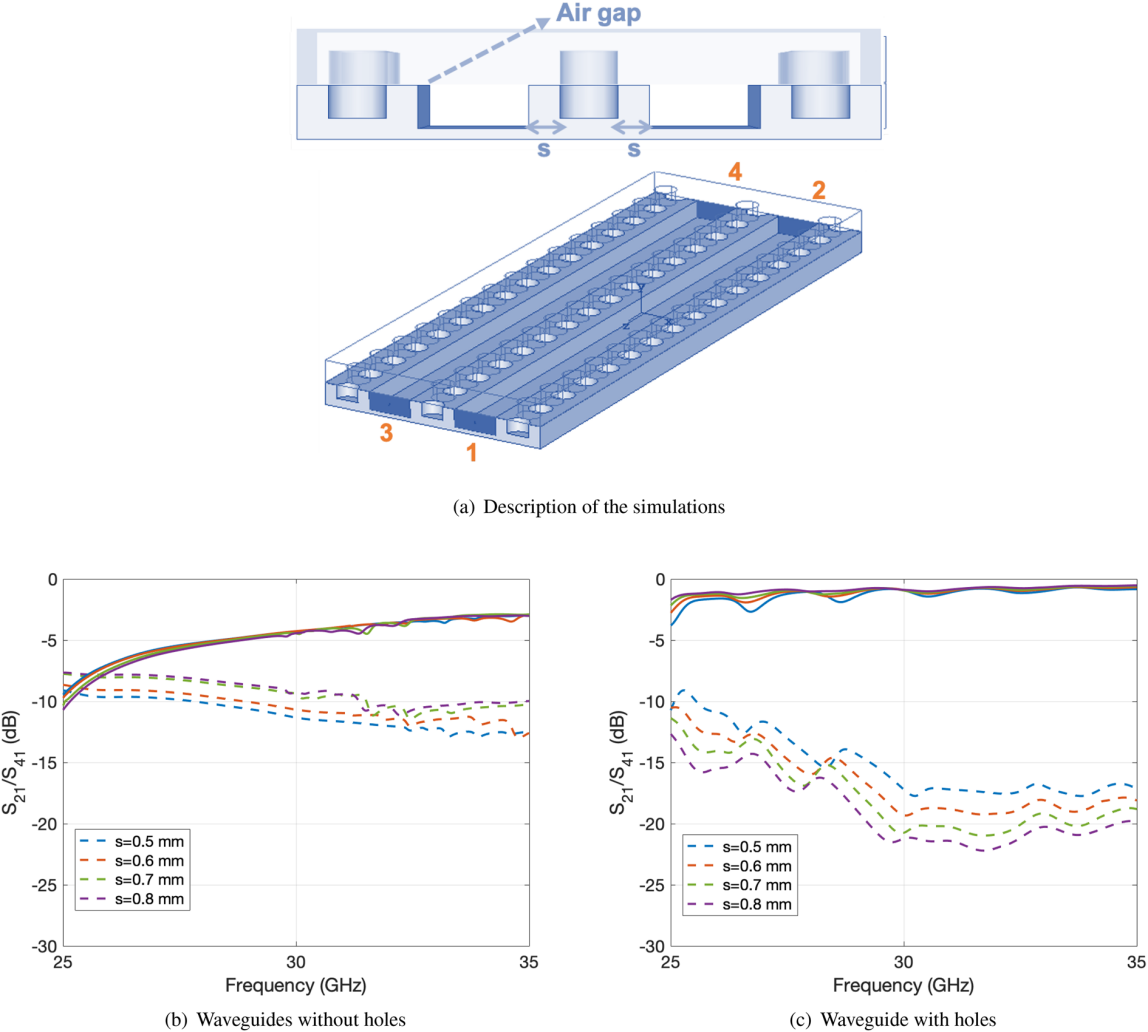
Simulated mutual coupling between contiguous waveguides (dashed lines) and transmission coefficient (solid lines) for different values of *s* for a waveguide with a gap of 0.1 mm.

The distance from the holes to the waveguide lateral wall *s* Fig. 5 has been set to 0.53 mm that is a trade off between the reduction in the coupling between contiguous waveguides when this distance is increased and the limitation of the total inter-row distance for the array. In this way, the total distance is 10.3 mm which is smaller than $\lambda _0$ as desired. Figure 5 shows the comparison of the coupling between two waveguides with (Fig. 5c) and without (Fig. 5b) for different values of *s*. In the same graphs, together with the coupling (dashed lines), the transmission coefficient is also represented. The simulations correspond to the case with a gap of 0.1 mm and in all cases the matching is below $-20$ dB.

**Figure 6:**
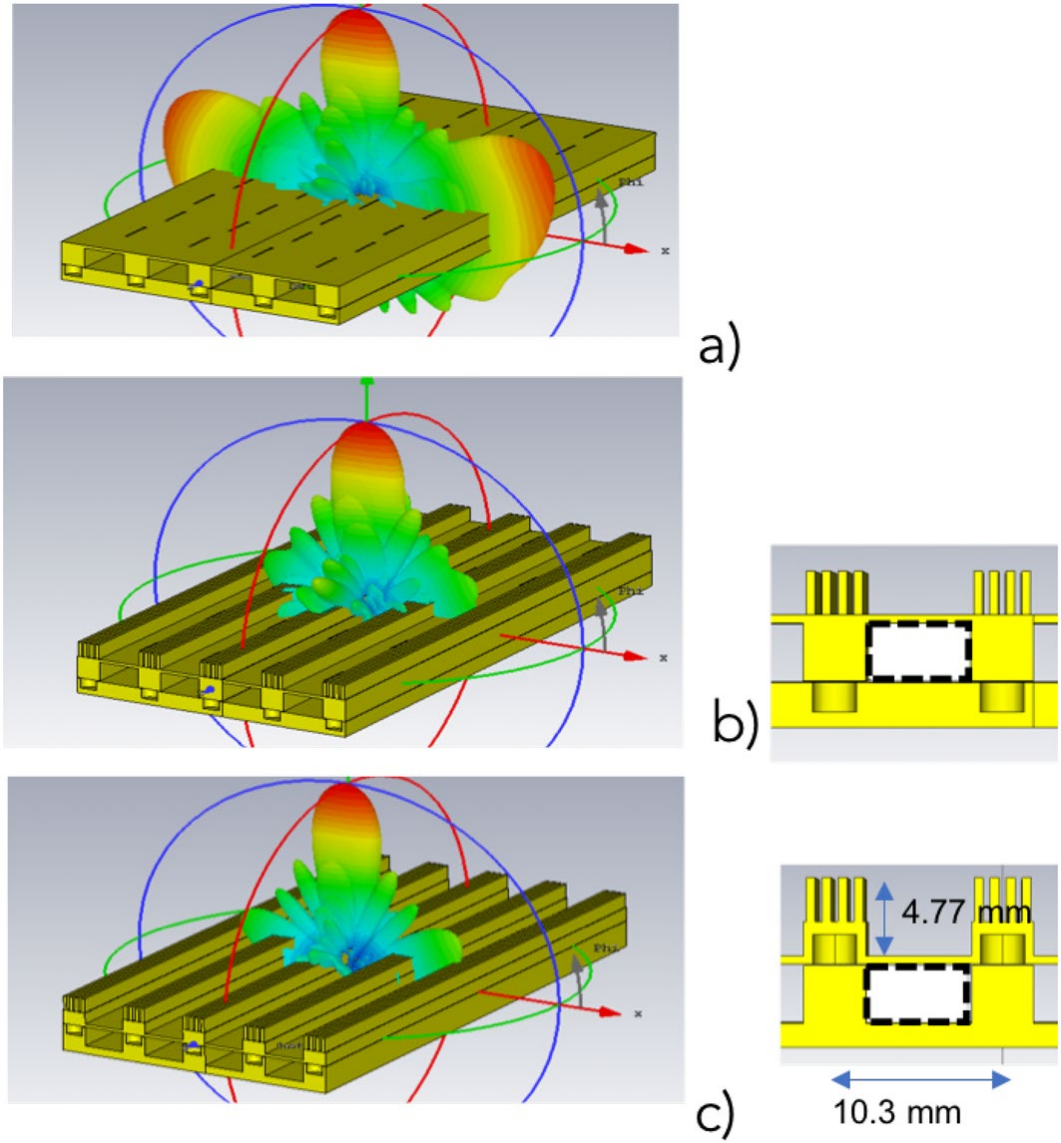
(**a**) Planar slotted array, (**b**) Planar slotted array with corrugations. (**c**) Planar slotted array with modified corrugations. All shown 3D radiation patterns correspond to 28 GHz.

The initial designed array is shown in Fig. 6a, for an example made with the four rows of slots. A directivity of 19.82 dBi is obtained but the side lobe level (SLL) in the E-plane is unacceptable as it can be seen in the figure due to the inter-row distance being too close to $\lambda _0$ and the omni-directional characteristic of the radiation pattern of the slot in that plane. Particularly, the SLL in the E plane is $-2.8$ dB whilst in the H-plane a $-12.5$ dB is obtained. All these results correspond to 28 GHz.

A solution to the problem with the SLL in E-plane was to add corrugations as shown in Fig. 6b as previously presented in^31^. In this case, the corrugations have a depth of 2.68 mm a periodicity of 0.98 mm and a width of 0.54 mm. In this figure, the reduction of the SLL is clearly visible. This has as consequence an increase in the antenna directivity up to 24.02 dBi and the SLL in E-plane is now reduced to $-14.7$ dB whilst in H-plane that level is $-12.7$ dB.

With the purpose of a simplest integration with the power divider as well as an easier manufacturing, a new version of the array with corrugations was studied and it is proposed in Fig. 6c. The main difference comes from how the holes and the waveguide are integrated. The performance is quite similar to the case in Fig. 6b. A directivity of 24.4 dBi is obtained and for this case the SLL in E-plane is $-15.8$ dB whilst in H-plane that level is $-13.8$ dB.

All the presented data of directivity and SLL in this section correspond to a frequency of 28 GHz.

## Experimental results

**Figure 7:**
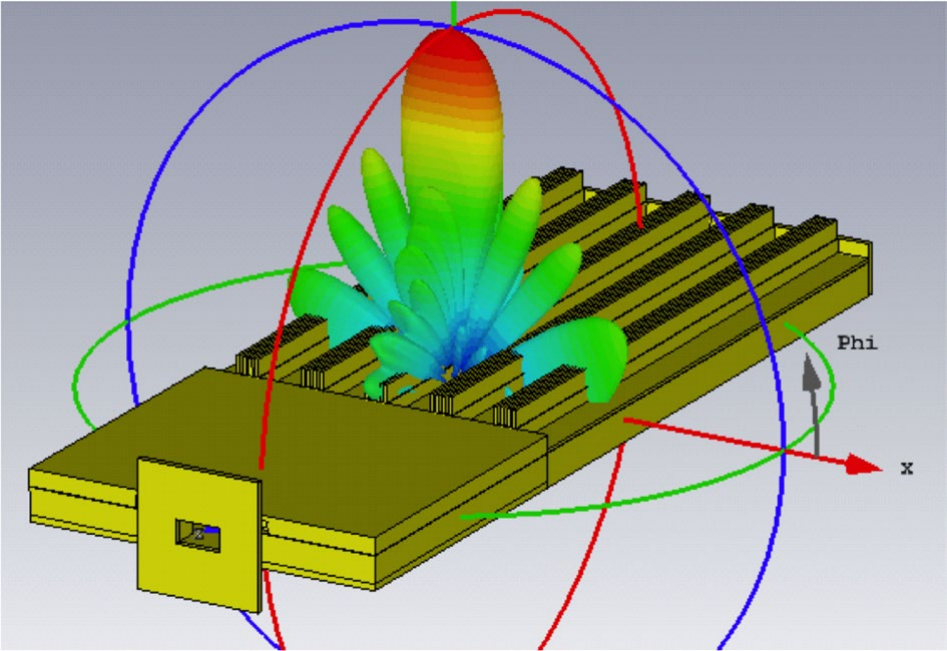
Simulated radiation pattern of the final manufactured prototype including antenna and feed network at 28 GHz.

**Figure 8:**
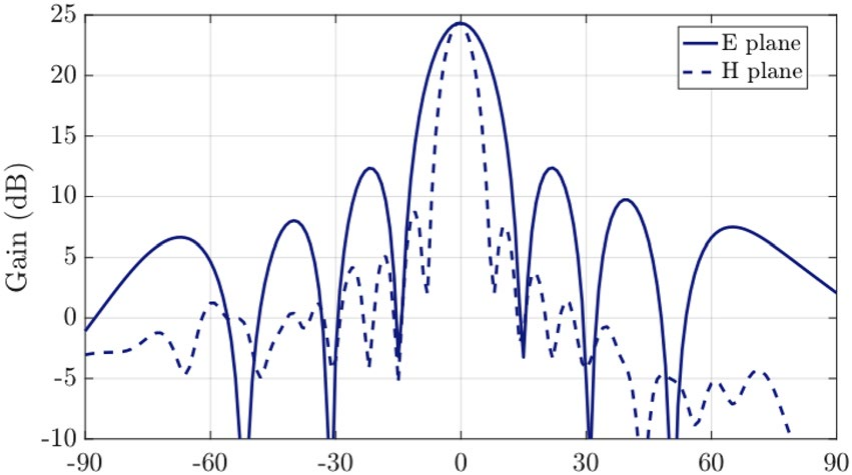
Simulated radiation pattern of the final manufactured prototype including antenna and feed network at 28 GHz.

A prototype has been manufactured and measured. Previously, the design was modified to incorporate the flange for the waveguide transition and the screws to assemble the two pieces together. The simulation results as 3D radiation pattern together with the final structure are shown in Fig. 7. The detailed radiation pattern in the two main planes at 28 GHz is presented in Fig. 8.

**Figure 9:**
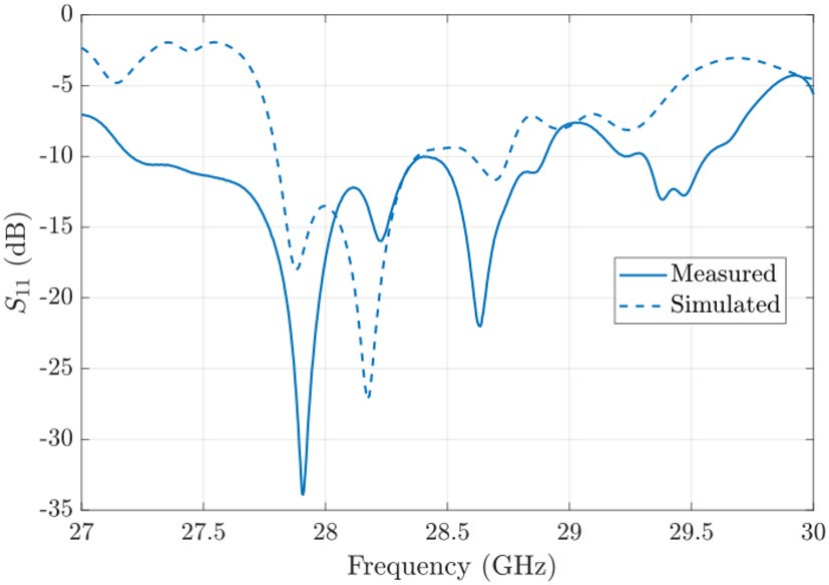
Simulated and measured $S_{11}$ parameter of the final prototype.

The simulated antenna input reflection coefficient is shown in Fig. 9. A good matching is observed from 27.5 to 29 GHz approximately. The slightly extended bandwidth that is observed when cmpared to simulations is probably due to some additional losses in the connection of the waveguide feed. The simulated directivity is 24.75 dBi at 28 GHz and the simulated realized gain at the same frequency is 24 dBi. Figure 12 includes a representation of the variation of the simulated directivity and realized gain as a function of the frequency (from 27.5 to 29 GHz).

**Figure 10:**
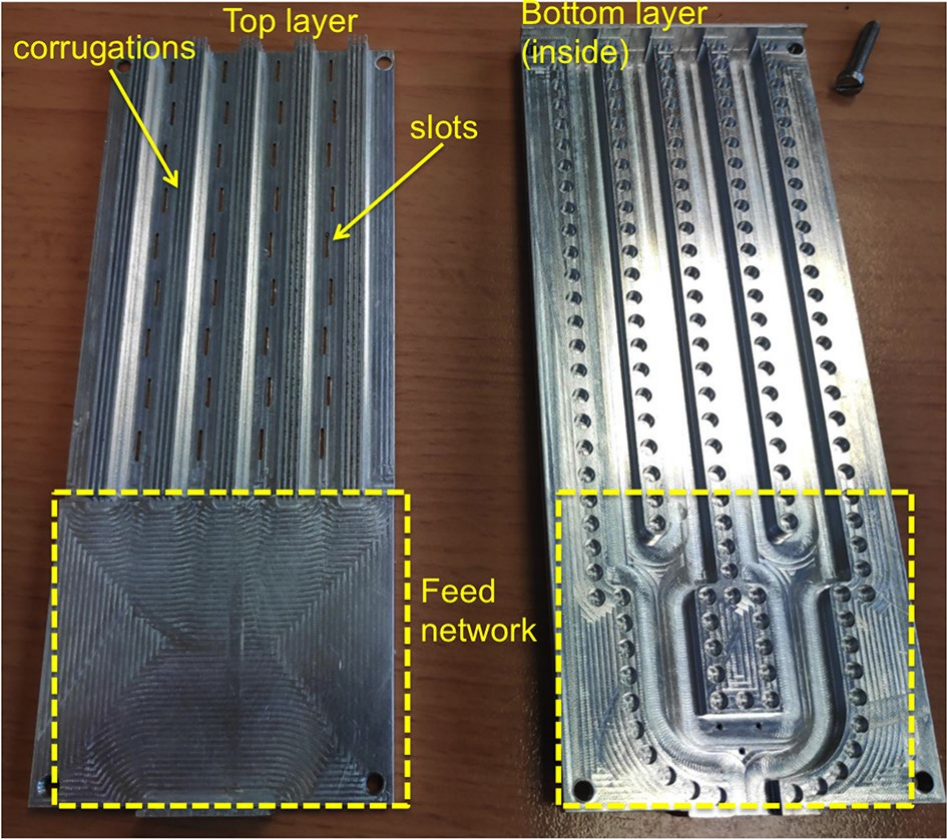
Picture of the two pieces of the fabricated antenna.

The manufactured prototype separated in two different layers can be seen in Fig. 10. The two pieces are simply assembled by using the four screws in the coners that can be seen in the picture. The total size of the prototype is 55 mm width by 135 mm length (45 mm correspond to the feed network and 90 mm to the antenna.) The measurement results in terms of antenna matching are presented in Fig. 9 where a good agreement between simulations and measurements is observed.

**Figure 11:**
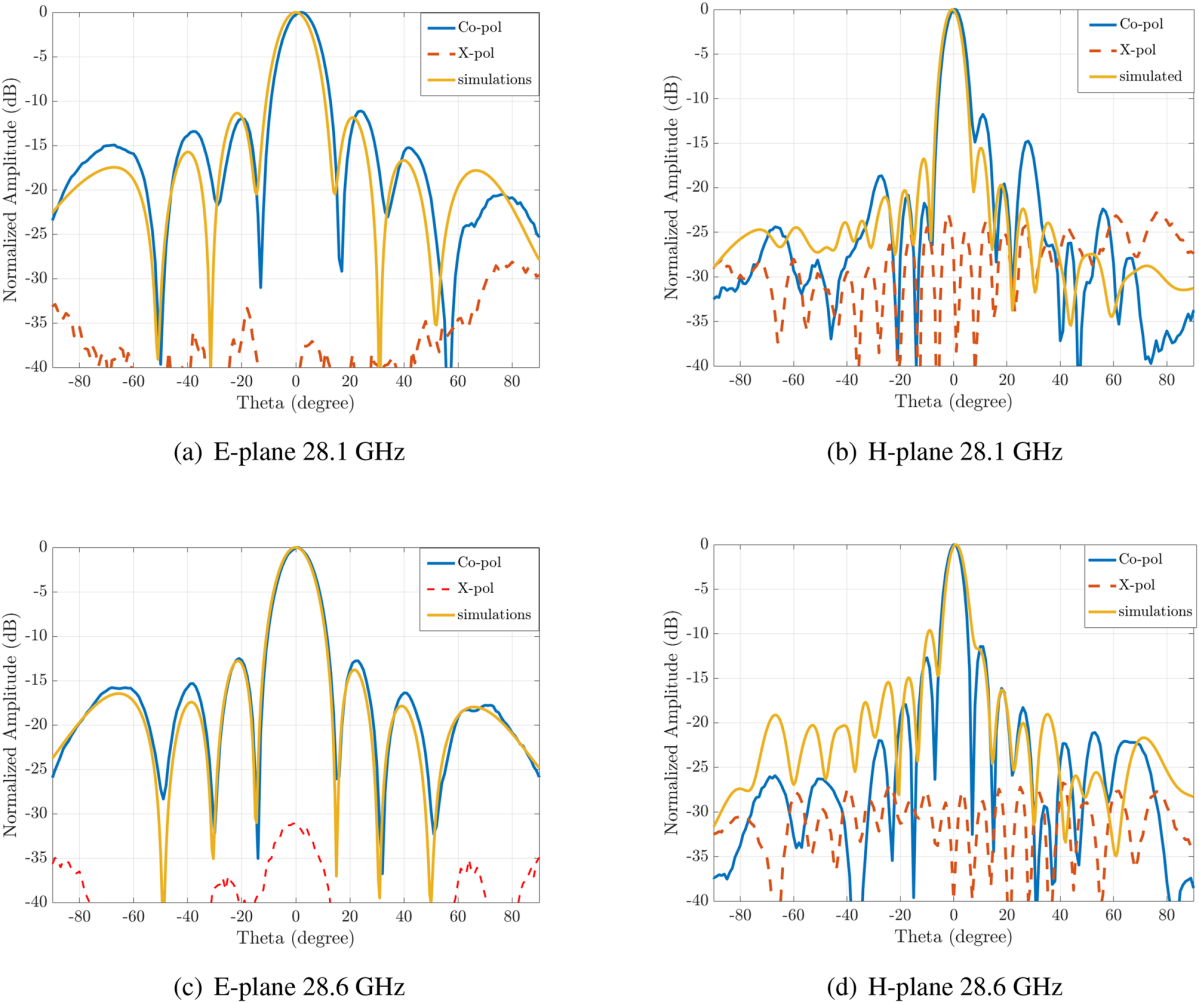
Measured E-plane and H-plane radiation patterns at the frequency of the maximum realized gain at two frequencies. In yellow the simulated results.

Finally, the measured radiation patterns at different frequencies in the two main plains and for two frequencies (28.1 and 28.6 GHz) can be seen in Fig. 11. Both the SLL and the antenna directivity are in good agreement with the simulations. Moreover, the antenna exhibits very low cross-polarization levels (around $-30$ dB).

**Figure 12:**
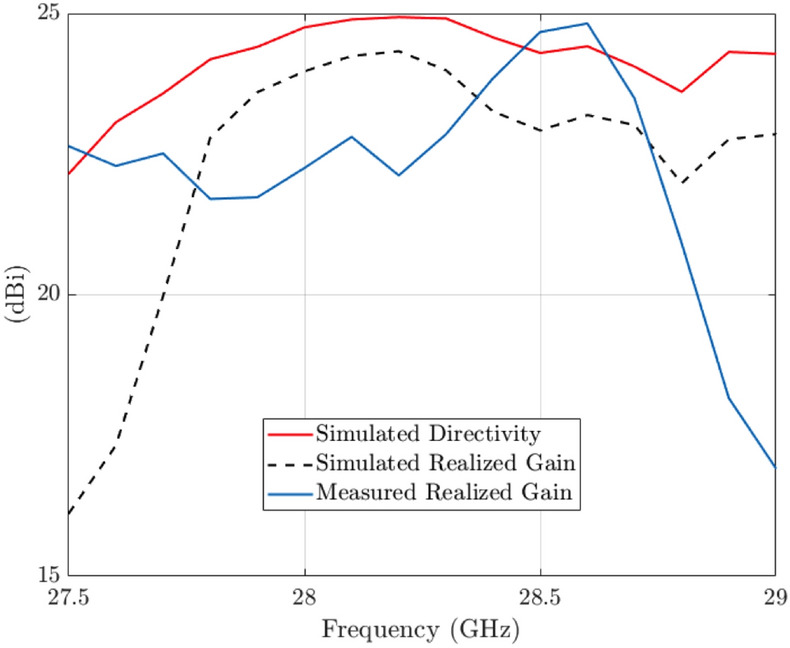
Comparison of the measured realized gain with the simulated realized gain and directivity as a function of the frequency.

The measured realized gain as a function of the frequency is presented in the last figure (Fig. 12) where also the simulated realized gain and directivity are included. The observed discrepancies are mainly due to the manufacturing tolerances. After observing this graph, we can conclude that probably caused by manufacturing tolerances, the maximum gain is shifted up in frequency. A supposition is that the slots are slightly smaller than the simulated ones. The maximum measured gain is in any case below the maximum simulated one. The antenna has a narrow band behaviour as it is expected for a slotted waveguide array, still there are applications where this bandwidth will be sufficient.

## Conclusion

The use of glide-symmetric EBG holes to design groove gap waveguide slot arrays is discussed in this paper. The holey unit cell size can compromise the inter-element distance of the rows of slots used in the planar slot arrays. We have explored a solution to overcome this issue that consists of limiting the size of the holes reducing the size of the stopband, using non standard dimensions for the waveguide as well as the use of corrugations in between the array rows to attenuate the grating lobes that appear at grazing direction when the inter-element distance is close to one wavelength. The latter was evaluated for different implementations.

A 1 to 4 compact power divider has been designed to feed this array with uniform amplitude and phase. The simulated and measured results are in good agreement and the feasibility of applying glide-symmetric holes to design groove gap waveguide slot arrays has been demonstrated showing a simpler solution in terms of manufacturing and assembly compared to the use of pins as unit cell.

Finally, a table comparing this work with other arrays in similar frequencies based on the use of single layer slotted waveguides is presented in Table 1. This includes: slotted waveguide in groove gap waveguide (GGWG) implemented with glide symmetric holes^21^, in GGWG with pins^16^, in conventional rectangular waveguide (RWG)^32^ and in SIW^33^. In the table we do not include the results of relative bandwidth as all the examples are narrow band and only the value of the impedance bandwidth is available, which is not representative enough for this kind of antennas as beam splitting with frequency happens. For reference^32^, the * indicates that the available data is the aperture efficiency instead of the radiation efficiency.

**Table 1:** Comparison with other slotted waveguide arrays.

	$f_0$ (GHz)	Element number	Size ($\lambda _0$)	Gain (dBi)	SLL (dB)	Rad. eff. ($\%$)	Array type
Liao et al.^21^	28	$4 \times 4$	$1.5 \times 1.5$	19.6	$-$ 13	78	GGWG
Shaterian et al.^16^	14	$10 \times 1$	$4.5 \times 0.15$	15	$-$ 27	93	GGWG
Park et al.^32^	25.3	$20 \times 16$	$18.3 \times 14.8$	30.5	$-$ 14.7	46*	RWG
Liu et al.^33^	31.5	$8 \times 8$	$14.7 \times 13.6$	18.74	$-$ 11.02	NA	SIW
This work	28.6	$4 \times 9$	$4.3 \times 8.6$	24.8	$-11.5$	75	GGWG
